# Cyclic pamidronate treatment for osteogenesis imperfecta: Report from a Brazilian reference center

**DOI:** 10.1590/1678-4685-GMB-2018-0097

**Published:** 2019-04-25

**Authors:** Bruna Pinheiro, Marina B. Zambrano, Ana Paula Vanz, Evelise Brizola, Liliane Todeschini de Souza, Têmis Maria Félix

**Affiliations:** 1 Graduate Program in Child and Adolescent Health, Universidade Federal do Rio Grande do Sul, Porto Alegre, RS, Brazil; 2 Medical Genetics Service, Hospital de Clinicas de Porto Alegre, Porto Alegre, RS, Brazil

**Keywords:** Osteogenesis imperfecta, bone fracture, clinical features, pamidronate treatment, compliance

## Abstract

Treatment of moderate and severe forms of osteogenesis imperfecta (OI) with cyclic pamidronate at the Reference Center for OI Treatment in Southern Brazil was studied. A retrospective cohort study was conducted from 2002 to 2012. Data were obtained during inpatient (drug infusion) and outpatient care. Clinical data, including the presence of blue sclerae, dentinogenesis imperfecta, history and site of the fractures, biochemical data, including calcium, phosphorus, and alkaline phosphatase levels, were systematically collected. Bone mineral density (BMD) was measured using dual energy X-ray absorptiometry (DXA). Forty-five patients (26 females) were included in the study, and the age of the patients at the time of diagnosis ranged from 1 to 144 months, with a median age (p25-p75) of 38 (5-96) months. Most cases presented OI-4 (51.1%), and the median age of the patients at the start of treatment was 3.3 years (25-75 percentiles: 0.5 - 8.7 years). Twenty-four patients (54.5%) had some adverse events or intercurrences during treatment, and the treatment compliance mean was 92.3% (± 10.7). The treatment with intravenous pamidronate has shown to be safe, well-tolerated, and effective in regard to the improvement of BMD and the reduction of the number of fractures in children and adolescents with OI.

## Introduction

Osteogenesis imperfecta (OI) is a heterogeneous group of connective tissue disorder that primarily affects bone, resulting in fragility and susceptibility to fractures by mild or no trauma. OI clinical features can vary from mild symptoms with a small number of fractures to severe short stature, bone deformities, and a great number of fractures to neonatal lethality (Martin and Shapiro, 2007; Shapiro and Germain-Lee, 2012; Van Dijk and Sillence, 2014). The prevalence of OI is 6 to 7 cases per 10,000 births, without ethnic distribution (Martin and Shapiro, 2007; Shapiro and Germain-Lee, 2012; Brizola *et al.*, 2016). Eighty-five to 90% of OI cases are caused by autosomal dominant inheritance mutations in *COL1A1* or *COL1A2* genes encoding collagen type 1, the major structural protein in bones, tendons, and ligaments. Additional research has uncovered rare autosomal recessive or X-linked mutations in the other 19 genes also involved in collagen biosynthesis or osteoblast function (Van Dijk and Sillence, 2014; Bregou-Bourgeois *et al.*, 2016; Thomas and DiMeglio, 2016).

Due to a large phenotypic variability of the disease, Sillence *et al.* (1979) suggested the first classification of OI into four types based on clinical and radiological criteria. Subsequently, other types, based primarily on molecular genetics, have been described. The working group *Nosology and Genetic Classification of Skeletal Disorders* suggested use of the original classification, adding Type 5 of OI (Bonafe *et al.*, 2015). Type 1 (OI-1) is non-deforming, characterized by none or few fractures; OI Type 2 (OI-2) is characterized by severe bone fragility and neonatal mortality; OI Type 3 (OI-3) is a severe form leading to multiple fractures, even in utero, and bone deformities; OI Type 4 (OI-4) is a moderate type with high clinical variability, characterized by fractures and variable stature; and OI Type 5 (OI-5) is a moderate form with the formation of hyperplastic callus in fracture sites, calcification of the interosseous membrane between tibia/fibula and radius/ulna, and dislocation of the radial head (Glorieux *et al.*, 2000; Brizola *et al.*, 2015). Due to fractures and other clinical characteristics, the management of OI is very complex. To date, there is no cure for OI; however, having an experienced multidisciplinary team of pediatricians, orthopedic surgeons, nurses, physiotherapists, and nutritionists ensures better prognosis, optimized mobility, and successful integration of patients into society (Cheung and Glorieux, 2008; Zambrano *et al.*, 2014; Marr *et al.*, 2017). Traditionally, treatment of OI involved orthopedic surgery and therapy; however, the severity of the disease led to pharmacological intervention aimed to decrease bone fragility (Glorieux, 2008).

In 1998, the use of intravenous bisphosphonate—sodium pamidronate (PD)—was first published to treat moderate to severe OI, showing increased bone mass, reduced fractures and bone pain, and improved mobility and quality of life (Glorieux *et al.*, 1998). Additional studies with other types of bisphosphonates (BPP) have been published since (Aström *a*nd Söderhall, 2002; Roughley *et al.*, 2003; Cheung and Glorieux, 2008; Alcausin *et al.*, 2013).

In 2001, the Brazilian Ministry of Health approved Reference Centers for OI Treatment, including the Hospital de Clinicas de Porto Alegre (HCPA) in Rio Grande do Sul State in Southern Brazil. The treatment of OI with PD was established as a public health policy and is covered by the Unified Health System, and the treatment protocol was updated in 2013 to introduce disodium alendronate as an alternative treatment (Lima and Horovitz, 2014).

The aim of this study was to evaluate children clinically diagnosed with OI (Types I, III, IV, or V) who were treated with cyclic sodium pamidronate and correlated these data to fractures, mobility, compliance to treatment, and adverse events in the Reference Centers for OI Treatment of HCPA.

## Subjects and Methods

### Subjects

One hundred twenty individuals of all ages from 87 unrelated families have been registered at the Reference Center for Osteogenesis Imperfecta Treatment at the Hospital de Clinicas de Porto Alegre (CROI-HCPA). All registered patients who received PD treatment from January 2002 to December 2012 were enrolled in this study, and each of these cases was reviewed retrospectively. The patient cohort included male and female children and adolescents (range 0-18 years) with clinical and radiological OI diagnoses. Cases with more than 50% of medical notes missing were excluded. This study was approved by the Research Ethics Committee of the Hospital de Clinicas de Porto Alegre (# 13-0079).

### Treatment

PD cycles were administered intravenously for three days during hospitalization. Doses of PD and the time interval varied according to the age of the patient. Children under two years of age received 0.5 mg/kg/day every two months. Patients aged two-to-three years received 0.75 mg/kg/day every three months. Patients older than three years received 1.0 mg/kg/day every four months. Each dose was diluted in 0.9% saline solution and infused intravenously for 3-4 h. During PD treatment, a calcium-rich diet was provided and supplemented with 1000 mg calcium carbonate and 400 to 800 IU of vitamin D. Antipyretics were prescribed for patients presenting fever or flu-like symptoms.

### Clinical features

Clinical data, including age, weight, height, number of fractures, blue sclerae, dentinogenesis imperfecta, family history of OI, and mobility, were recorded before and during the treatment at the outpatient OI clinic. Anthropometric measurements were performed according to standard procedures: weight was measured using digital electronic scales for babies (Filizola® Baby, São Paulo, SP) and children (Filizola® Personal, São Paulo, SP), and height was measured using a stadiometer in standing position (Filizola® Personal, São Paulo, SP). For children and adolescents unable to stand due to the severity of disease, height was measured in the supine position. If there was a difference between the lengths of the lower limbs, the longest was measured. All data were calculated according to anthropometric z-scores for weight and height using licensed software (Multicentre WHO *et al.*, 2006). Patient mobility measurement was collected at the first and last medical visit according to the Land *et al.* (2006) mobility score criterion system: (0) restricted to wheelchair; (1) able to walk with assistance; (2) able to walk at home with or without assistance; (3) able to walk short distances with or without assistance; and (4) able to walk independently. Infants and children under one year of age were excluded from the mobility analysis.

The incidence of fractures was evaluated during follow-up visits. Data were recorded using a combination of parental recall, radiographic exams, and medical records. The fracture rate was determined by the number of fractures at baseline compared to the number of fractures per year during the period that the patient was receiving PD treatment. Non-dislocated fissures, rib fractures, and refractures—fractures occurring at least partially along the previous fracture line within a year or before its full ossification—were not included.

### Biochemical data

Biochemical analyses were performed during cyclic infusion. Samples were collected on the first day of hospitalization prior to infusion and on the third day after the end of the cycle. Parameters, such as calcium, phosphorus, and alkaline phosphatase (ALP), were collected systematically. Total calcium, phosphorus, and ALP were analyzed using molimetric, UV phosphomolybdate, and kinetic methods, respectively (Roche Hitachi 917; Advial 1800).

### Bone densitometry

Bone mineral density (BMD) in the spine (L1-L4) and the total body were calculated using the dual energy X-ray absorptiometry method (DXA) (QDR HOLOGIC - 4500, 26th version 8: 3; Bedford, MA, USA). A z-score below two standard deviations for what was expected according to chronological age of the patient was defined as low BMD, as defined by the official consensus of Brazilian Society of Clinical Densitometry in 2006 (Zerbini *et al.*, 2007). DXA was performed preferentially once a year for each patient; however, some cases did not follow this recommendation due to local limitation.

### Treatment compliance

Compliance to treatment was assessed considering the number of cycles established for each patient, according to the protocol, divided by the number of cycles carried out. Good compliance was assigned when 80% of cycles or more were performed. The reasons for absences, including unexcused absence, surgery, and fracture, were recorded.

### Statistical analyses

Quantitative variables were expressed as mean and standard deviation, or median and interquartile range, and categorical variables were described as absolute and relative frequencies. To compare means before and after treatment, Student’s *t*-test was applied. To compare fracture rates at baseline, one year of treatment and after, the Log Rank test was applied, and to compare the number of fractures per month by the type of OI, Kruskal-Wallis, and Mann-Whitney tests were used. Association between the type of OI and compliance was evaluated by one way Analysis of Variance (ANOVA), and associations between continuous variables with asymmetric distribution were evaluated by Spearman’s correlation coefficients. A comparison of serum ALP and BMD over time was evaluated by the model of generalized estimating equations adjusted with Bonferroni correction. The significance level was 5%. All analyses were performed using SPSS software (ver. 18.0; SPSS Inc, Chicago, Illinois).

## Results

During the study period, 48 patients with OI were treated with PD. Of these, three patients were excluded from analysis due incomplete medical records. The sample consisted of 45 patients total, 26 of female gender. Of those 45; 34 (75.6%) underwent molecular analysis, and mutations were identified in the *COL1A1*, *COL1A2*, and *IFTIM5* genes in 21(61.8%), 11 (32.4%), and 2 (5.9%) cases, respectively. Due to the small number of individuals with OI–5, these were grouped with OI–4, considering that both types represent moderate forms of OI. The age of the patients at the time of diagnosis ranged from 1 to 144 months, with a median age (p25-p75) of 38 (5-96) months. Clinical data are shown in Table 1. Most of the patients were classified with OI-4 (51.1%), followed by OI-1 (22.2%) and OI-3 (22.2%). Only two patients were classified as OI-5 (4.5%). The average age of the patients at the first pamidronate treatment was 3.3 years (25-75 percentiles 0.5-8.7 years), the median number of cycles was 10 (25-75 percentiles: 7-13 cycles), and the median of dose per cycle was 49.9 mg (percentiles 25-75: 25-83.3 mg). Positive family history of OI was observed in 30 patients (66.7%). In two cases, family history was unknown because the children were adopted. Of all patients, 44 (97.8%) had blue sclerae, and, of 32 patients who presented teeth during the first evaluation, 13 (40.6%) had dentinogenesis imperfecta.

**Table 1:** Clinical data of the sample

Variables	n = 45
Gender – n(%)	
Male	19(42.2)
Female	26(57.8)
Family history of OI –n (%)	30(66.7)
Mutation analysis	34(75.6)
COL1A1 gene	21(61.8)
COL1A2 gene	11(32.4)
IFITM5 gene	2(5.9)
OI type – n(%)	
1	10(22.2)
2	10(22.2)
3	23(51.1)
5	2(4.5)
Blue sclerae- n(%)	44(97.8)
Dentinogenesis imperfecta –n = 32(%)	13(40.6)
Age at first pamidronate cycle treatment (years) –md(P25-P75)	3.3(0.5-8.7)
Number of pamidronate cycles –md(P25-P75)	10(7-13)
Dose/cycle(mg) –md(P25-P75)	49.9(25-83.3)

md median

**Figure 1:**
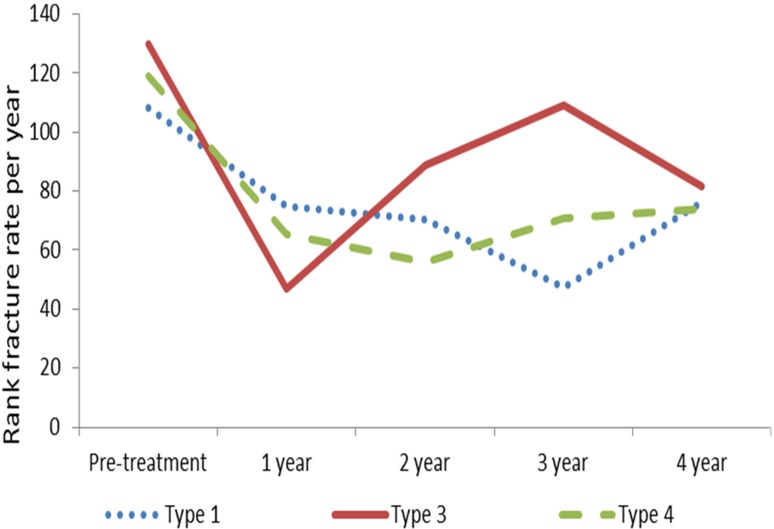
Fracture rate in the sample and according to OI type.

The fracture rate (Figure 1) showed a different response to treatment according to OI type. There was a significant correlation between the type of OI and time of treatment (p < 0.001). Individuals with OI-1 had a significant reduction in the number of fractures in the third year of treatment with pamidronate (p = 0.032). For those with OI-3, the behavior of the fractures had a variation, with a decrease in the first year of treatment, an increase in the rate in the 2nd and 3rd years, and a decrease in the 4th year (p < 0.001). Patients with OI-4 had also a reduction in fractures (p < 0.001). With OI-1 and -3, the differences are significant between the initial and final assessments, and, in OI-4, the differences are significant between pretreatment and the first year of treatment.

In our sample we observed that cases that started the treatment later had an increase in the number of fractures over time compared to the baseline. This behavior was observed only in Types 3 (rs 0.78, p < 0.010) and 4 (rs > 0.49, p < 0.050).

Table 2 shows that there was a significant difference between the site of fractures and the OI type. During treatment, the majority of patients (n = 27; 69.2%) had fractures in their femurs, followed by radius/ulna (n = 23; 59%). A fracture of the tibia occurred in 80% of the individuals with OI-1, differing from OI-3, which presented umerus fractures in 90% of cases, and OI-4, which presented most of their fractures (73.7%) in the femur. No atypical femur fracture was recorded in our sample.

**Table 2:** Number and site of fractures according to OI type.

Site of fractures	Total n(%)	OI -1(%)	OI-3(%)	OI-3 (%)	*p*
humerus	23 (59)	40	90	52.6	0.056
radius/ ulna	12 (30.8)	30	60	15.8	0.049
femur	27 (69.2)	50	80	73.7	0.293
tibia	20 (51.3)	80	40	42.1	0.108
spine	6 (15.4)	20	0	21.1	0.294

**Figure 2:**
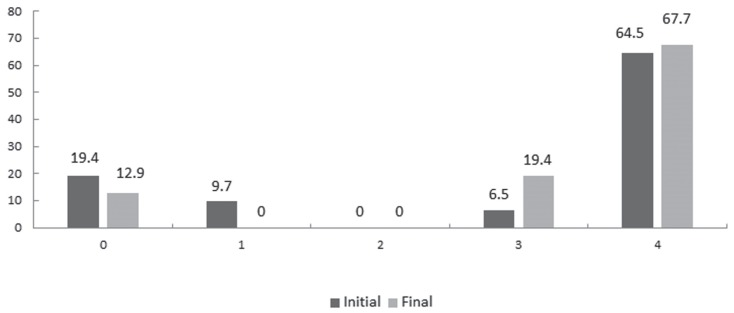
Mobility score criterion system (Land *et al.* (2006): (0) restricted to wheelchair; (1) able to walk with assistance; (2) able to walk at home with or without assistance; (3) able to walk short distances with or without assistance; and (4) able to walk independently.

**Figure 3:**
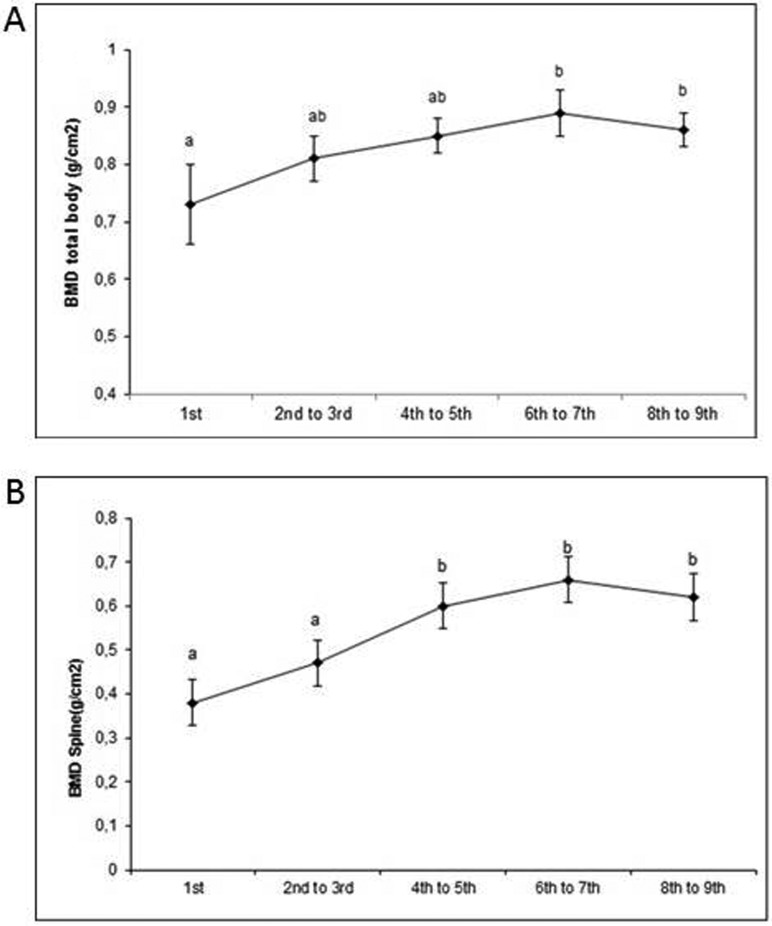
Bone mineral density of the whole body (A) and of the spine (B) by OI type. a b Does not differ by Bonferroni test at 5% significance (GEE).

Thirty-one patients were evaluated according to the Land *et al.* (2006) criteria for mobility (Figure 2). Although no significant difference was observed, there is a tendency of improvement. There was a decrease in the number of patients restricted to a wheelchair, and we observed an increase in the group of patients who were able to walk short distances with or without help. Figure 3 shows that, regardless of OI type, the BMD of the total body (less head) increased from one-to-six years (p < 0.001). In the lumbar spine, an improvement was observed after the fourth year of treatment (p < 0.001).

The average calcium level prior to each infusion of PD was 9.58 ± 0.58 mg/dL, and, at the end of the cycle, was 8.94 ± 0.62 mg/dL (p < 0.001). The average phosphorus was 5.07 ± 0.66 mg/dL at the beginning of each cycle and 4.5 ± 0.66 mg/dL at the end (p < 0.001). Additionally, there was a decrease in ALP from the first cycle (510.57 U/ L) to the last (203 U/L, p < 0.001).

Twenty-four patients (54.5%) had some form of adverse event during treatment: fever was recorded in 23 (95.8%) subjects, vomiting and influenza-like illness in 3 each (12.5% each), and asymptomatic hypocalcemia in 1 (4.2%). Most of the adverse events occurred at the first exposure to intravenous PD. Respiratory distress syndrome, symptomatic hypocalcemia, or fatal events were not reported during PD treatment.

The mean of compliance to treatment was 92.3% (± 10.7). Considering good compliance as a percentage equal to or above 80%, 39 patients (86.7%) adhered to treatment, and, of the total sample, 26 patients (57.8%) completed the treatment. The reasons for treatment absence for 19 patients were fracture (*n* = 2), surgery (*n* = 2), and H1N1 influenza (*n* = 1). Seven patients missed treatments without justification, and the rest of the patients presented more than one reason. No significant differences were observed in compliance between different types of OI (p = 0.377). There was a significant positive association between compliance to treatment and the number of fractures per year (rs = 0.319, p = 0.033). When stratified by type of OI, an association was observed with OI-3 (rs = 0.623, p = 0.054) but not OI-1 (rs = 0.154, p = 0.671) nor OI-4 (rs = 0.214, p = 0.328).

## Discussion

As OI is a rare disorder, treatment at a center with experience is required. This study describes the experience of treatment with sodium pamidronate in 45 patients with OI attending a Reference Center for OI Treatment in Southern Brazil. Our data showed variable improvement during treatment with pamidronate according to OI type.

Intravenous bisphosphonate therapy is broadly used to treat bone fragility in children with OI. In all types of OI, a decrease in the number of fractures was observed throughout the treatment when compared to baseline. However, our study showed that the behavior of the fractures differed with respect to OI type. In OI-3, a reduction of fractures was observed in the first year of treatment, but, in the following years there was an increase in occurrence. Bisphosphonates have been widely used for the treatment of OI in routine clinical practice in both adults and children, but the evidence base for fracture prevention is still limited (Hald *et al.*, 2015). Recent studies with different types of bisphosphonates are controversial in relation to reduction in the rate of fractures in OI (Letocha *et al.*, 2005; Chevrel *et al.*, 2006; Rauch *et al.*, 2009; Ward *et al.*, 2011; Bishop *et al.*, 2010).

[Bibr B35] Palomo *et al.* (2015) analyzed the results of treatment with pamidronate in 37 children with OI who started BPP treatment before 5 years of age and had a subsequent follow-up period of at least 10 years. Although long-term pamidronate therapy was associated with higher z-scores for spine BMD and for vertebral remodeling, the rate of long bone fracture remained high, and most patients developed scoliosis.

A hypothesis that may explain this finding is that OI-3 patients were diagnosed earlier than the other types due to the clinical severity. As treatment continues, pain and mobility improve, leading to achieve motor development milestones (sitting, standing, gait), resulting in a greater chance of fractures. In addition, it is known that the marked increase in bone fragility in OI is caused not only by low bone density but also by the abnormal bone matrix. This differs from the situation in osteoporosis, where the bone matrix is normal, but bone density is reduced and bisphosphonates have beneficial effects in preventing fractures (Sambrook and Cooper, 2006; Hald *et al.*, 2015).

Bone fragility and susceptibility to fractures with no or minimal trauma are typical of OI features. In a previous study of our group, for mild OI cases, it was observed that initial fractures tend to occur during the period in which children start to walk because the upright posture promotes increased weight load on the lower limbs, leading to secondary fractures (Brizola *et al.*, 2014).

The ability of intravenous PD to decrease fracture rates during treatment has been demonstrated in several studies (Glorieux *et al.*, 1998; Plotkin *et al.*, 2000; DiMeglio *et al.*, 2004; Forin *et al.*, 2005; Alcausin *et al.*, 2013; Kusumi *et al.*, 2014). A significantly decreased fracture rate in upper extremities (p = 0.040) but not in lower extremities (p = 0.090) after one year of intravenous PD treatment was observed in an American study (DiMeglio *et al.*, 2004). After two years of treatment, the fracture rate did not decrease further in the upper (p = 0.840) or lower extremities (p = 0.290). Two systematic Cochrane reviews were not conclusive regarding the use of bisphosphonates to significantly reduce the incidence of fractures in patients with OI (Philippi *et al.*, 2008; Bachrach and Ward, 2009). Recent systematic reviews focused on the use of bisphosphonates and prevention of fractures did not provide support for this hypothesis (Dwan *et al.*, 2016; Rauch *et al.*, 2017), emphasizing the need for appropriate randomized studies to evaluate fracture rates and the risks and benefits of using bisphosphonates. Continued monitoring of OI patients is also important, and it is necessary to address fracture rates after stopping treatment.

The increases in BMD recorded here were consistent with previous reports (Aström and Söderhall, 2002; Rauch *et al.*, 2003; Arikoski *et al.*, 2004; DiMeglio *et al.*, 2004; Lindahl *et al.*, 2016). Letocha *et al.* (2005) observed that children of the age of 4 to 13 years treated with sodium pamidronate showed an increased BMD, volume, area, and height of the L1-L4 vertebrae.

Efficacy was demonstrated in individuals by improving BMD values, independent of OI type. We observed improvements in overall body BMD after the sixth year of treatment (p < 0.001) and in the spine after the fourth year (p < 0.001). The observed delay in improvement in BMD may be due to fact that many patients did not perform the DEXA annually.

In this study, we did not observe a significant improvement in the mobility of the patients after treatment. However, the small number of patients evaluated may have been a limiting factor in the analysis, since only 31 of the 45 children could be assessed by the Land criteria. Fourteen children were younger than one year at the beginning of treatment. This represents almost a third of our sample, which is positive considering that the earlier the treatment starts the greater the benefits for the child.

Increased mobility of children during treatment may be due to a combination of factors, such as improved BMD, fracture reduction, increased muscle strength, and decreased pain (Montpetit *et al.*, 2003; Letocha *et al.*, 2005; Land *et al.*, 2006). However, it is expected that functional capacity and improved mobility can also improve naturally as children grow and gain skills (Letocha *et al.*, 2005).

In the literature, there is no consensus on the bisphosphonate agent and dosage, as well as on the optimal duration of therapy in children (Plotkin *et al.*, 2000; Antoniazzi *et al.*, 2006; Alcausin *et al.*, 2013; Kusumi *et al.*, 2014). When comparing the results of these studies, it should be noted that differences in age and diagnoses of patients might influence responses to drug therapy, independent of dose or type of drug. The protocol used in this study was similar to a recent study describing the benefits of treatment with intravenous PD in children with OI under 24 months (Kusumi *et al.*, 2014). These authors observed that treatment with cyclic PD in children was safe and resulted in significant increases in BMD at the lumbar spine and reduced fracture rates. Another study compared 56 patients treated with PD to 167 patients who did not receive PD. In all patients receiving PD, there was an increase in BMD (p < 0.001) compared to patients who did not receive treatment. Patients with the greatest deficit in bone mass at the beginning of the study were those that had the greatest bone mass improvement during therapy (Rauch *et al.*, 2003).

After each cycle of PD administration, serum calcium and phosphorus levels decreased, similar to previously reported results (DiMeglio *et al.*, 2004). The initial mean ALP was higher than normal reference values before the first administration of PD, but after cyclical infusion, values were decreased. These results indicate that the rate of bone turnover decreases during the treatment, resulting in a balance between formation and resorption that promotes increased bone mass (Glorieux *et al.*, 1998).

Hypocalcemia, fever, and vomiting are well-known adverse events during the first cycle of intravenous bisphosphonate, such as pamidronate, ibandronate, or zoledronate (Trejo and Rauch, 2016). In our sample, the reported adverse events were fever, vomiting, and flu-like symptoms, each previously well described in the first infusion of PD, but controlled with the administration of antipyretics. These results are consistent with previous studies, suggesting that bisphosphonates are generally well tolerated in pediatric patients with limited side effects (Glorieux *et al.*, 1998; Eghbali-Fatourechi, 2014). Severe adverse events were not observed during treatment with cyclic PD. Recent research has evaluated the relationship between prolonged use of bisphosphonate and the incidence of atypical femur fractures (Lim *et al.*, 2016; Trejo *et al.*, 2017). In our sample, no patients had atypical femoral fractures during the PD treatment period.

Compliance to treatment was considered good, but several reasons for interruption of treatment were reported. Compliance, the measure of a person’s behavior according to the recommendations of a health professional, was measured. Although there is no standard measure to establish compliance, it is estimated that non-compliance rates for treatment for chronic diseases are high (Nalin *et al.*, 2010). To best of our knowledge, there is only one study that reported compliance to treatment in OI with PD. An Argentinian study in children with OI showed better quality of life scores with increasing treatment compliance (Fano *et al.*, 2013). In children with OI-3 and OI-4, there was a decrease in the quality of life in the physical domain, both in the perception of parents and children. The lower number of fractures, treatment with higher doses of pamidronate, and adherence to treatment were the variables related to a better quality of life in the severe forms of the disease. In our study, when the compliance was analyzed according to OI type, significantly higher differences were observed in individuals with the highest number of fractures per year, suggesting that patients with more severe disease are more adherent to treatment.

The present study has limitations. It is a retrospective study based on medical records, and some data, especially the number of fractures at diagnosis, were collected based on clinical histories and medical records. Furthermore, not all radiographs were reviewed by the authors. Another limitation was the measurement of BMD, where z-scores were not collected because there is no standard for children under five years in the study center, and the sample contained many children under five years old. Moreover, bone biomarkers, osteocalcin, parathyroid hormone, and N-telopeptide were not collected.

In conclusion, the first 10 years of treatment with cyclic intravenous PD proved safe and effective in reducing fractures (especially in the first few years of treatment), mobility, and BMD improvement. Adherence to treatment was also considered good in children and adolescents with OI and was inversely correlated to severity of the disorder.

## References

[B1] Alcausin MB, Briody J, Pacey V, Ault J, McQuade M, Bridge C, Sillence DO, Munns CF (2013). Intravenous pamidronate treatment in children with
moderate-to-severe osteogenesis imperfecta started under three years of
age. Horm Res Paediatrics.

[B2] Antoniazzi F, Zamboni G, Lauriola S, Donadi L, Adami S, Tatò L (2006). Early bisphosphonate treatment in infants with severe
osteogenesis imperfecta. J Pediatr.

[B3] Arikoski P, Silverwood B, Tillmann V, Bishop NJ (2004). Intravenous pamidronate treatment in children with moderate to
severe osteogenesis imperfecta: Assessment of indices of dual-energy X-ray
absorptiometry and bone metabolic markers during the first year of
therapy. Bone.

[B4] Aström E, Söderhäll S (2002). Beneficial effect of long term intravenous bisphosphonate
treatment of osteogenesis imperfecta. Arch Dis Child.

[B5] Bachrach LK, Ward LM (2009). Clinical review 1: Bisphosphonate use in childhood
osteoporosis. J Clin Endocrinol Metab.

[B6] Bishop N, Harrison R, Ahmed F, Shaw N, Eastell R, Campbell M, Knowles E, Hill C, Hall C, Chapman S (2010). A randomised controlled doseranging study of risedronate in
children with moderate and severe osteogenesis imperfecta. J Bone Miner Res.

[B7] Bonafe L, Cormier-Daire V, Hall C, Lachman R, Mortier G, Mundlos S, Nishimura G, Sangiorgi L, Savarirayan R, Sillence D (2015). Nosology and classification of genetic skeletal disorders: 2015
Revision. Am J Med Genet.

[B8] Bregou Bourgeois A, Aubry-Rozier B, Bonaf L, Laurent-Applegate L, Pioletti DP, Zambelli PY (2016). Osteogenesis imperfecta: From diagnosis and multidisciplinary
treatment to future perspectives. Swiss Med Wkly.

[B9] Brizola E, Staub ALP, Félix TM (2014). Muscle strength, joint range of motion, and gait in children and
adolescents with osteogenesis imperfecta. Pediatr Phys Ther.

[B10] Brizola E, Mattos EP, Ferrari J, Freire POA, Germer R, Llerena JC, Félix TM (2015). Clinical and molecular characterization of Osteogenesis
Imperfecta Type V. Mol Syndromol.

[B11] Brizola E, Félix TM, Shapiro JR (2016). Pathophysiology and therapeutic options in osteogenesis
imperfecta?: An update. Res Reports Endocr Disord.

[B12] Cheung MS, Glorieux FH (2008). Osteogenesis Imperfecta: Update on presentation and
management. Rev Endocr Metab Disord.

[B13] Chevrel G, Schott AM, Fontanges E, Charrin JE, Lina-Granade G, Duboeuf F, Garnero P, Arlot M, Raynal C, Meunier PJ (2006). Effects of oral alendronate on BMD in adult patients with
osteogenesis imperfecta: A 3-year randomized placebo-controlled
trial. J Bone Miner Res.

[B14] DiMeglio L, Ford L, McClintock C, Peacock M (2004). Intravenous pamidronate treatment of children under 36 months of
age with osteogenesis imperfecta. Bone.

[B15] Dwan K, Phillipi CA, Steiner RD, Basel D (2016). Bisphosphonate therapy for osteogenesis
imperfecta. Cochrane Database Syst Rev.

[B16] Eghbali-Fatourechi G (2014). Bisphosphonate therapy in pediatric patients. J Diabetes Metab Disord.

[B17] Fano V, Del Pino M, Rodríguez M, Buceta S, Obregón G (2013). Osteogénesis imperfecta?: Estudio de la calidad de vida en los
niños. Hosp Nac Pediatría.

[B18] Forin V, Arabi A, Guigonis V, Filipe G, Bensman A, Roux C (2005). Benefits of pamidronate in children with osteogenesis imperfecta:
An open prospective study. Joint Bone Spine.

[B19] Glorieux FH (2008). Osteogenesis imperfecta. Best Pract Res Clin Rheumatol.

[B20] Glorieux FH, Bishop NJ, Plotkin H, Chabot G, Lanoue G, Travers R (1998). Cyclic administration of pamidronate in children with severe
osteogenesis imperfecta. N Engl J Med.

[B21] Glorieux FH, Rauch F, Plotkin H, Ward L, Travers R, Roughley P, Lalic L, Glorieux DF, Fassier F, Bishop NJ (2000). Type V osteogenesis imperfecta: A new form of brittle bone
disease. J Bone Miner Res.

[B22] Hald JD, Evangelou E, Langdahl BL, Ralston SH (2015). Bisphosphonates for the prevention of fractures in osteogenesis
imperfecta: Meta-analysis of placebo-controlled trials. J Bone Miner Res.

[B23] Kusumi K, Ayoob R, Bowden SA, Ingraham S, Mahan JD (2014). Beneficial effects of intravenous pamidronate treatment in
children with osteogenesis imperfecta under 24months of age. J Bone Miner Metab.

[B24] Land C, Rauch F, Montpetit K, Ruck-Gibis J, Glorieux FH (2006). Effect of intravenous pamidronate therapy on functional abilities
and level of ambulation in children with osteogenesis
imperfecta. J Pediatr.

[B25] Letocha AD, Cintas HL, Troendle JF, Reynolds JC, Cann CE, Chernoff EJ, Hill SC, Gerber LH, Marini JC (2005). Controlled trial of pamidronate in children with types III and IV
osteogenesis imperfecta confirms vertebral gains but not short-term
functional improvement. J Bone Miner Res.

[B26] Lim H-S, Kim C-K, Park Y-S, Moon Y-W, Lim S-J, Kim S-M (2016). Factors associated with increased healing time in complete
femoral fractures after long-term bisphosphonate therapy. J Bone Jt Surg.

[B27] Lindahl K, Kindmark A, Rubin C, Malmgren B, Grigelioniene G, Söderhäll S (2016). Decreased fracture rate, pharmacogenetics and BMD response in 79
Swedish children with osteogenesis imperfecta types I, III and IV treated
with Pamidronate. Bone.

[B28] Lima MADFDD, Horovitz DDG (2014). Contradições das políticas públicas voltadas para doenças raras:
o exemplo do Programa de Tratamento da Osteogenese Imperfeita no
SUS. Cienc Saude Colet.

[B29] Marr C, Seasman A, Bishop N (2017). Managing the patient with osteogenesis imperfecta: A
multidisciplinary approach. J Multidiscip Health.

[B30] Martin E, Shapiro JR (2007). Osteogenesis imperfecta: Epidemiology and
pathophysiology. Curr Osteoporos Rep.

[B31] Montpetit K, Plotkin H, Rauch F, Bilodeau N, Cloutier S, Rabzel M, Glorieux FH (2003). Rapid increase in grip force after start of pamidronate therapy
in children and adolescents with severe osteogenesis
imperfecta. Pediatrics.

[B32] Multicentre WHO, Reference G, Group S (2006). WHO Child Growth Standards based on length/height, weight and
age. Acta Paediatr Suppl.

[B33] Nalin T, Perry IDS, Refosco LF, Netto CBO, Souza CFM, Picon PD, Schwartz IVD (2010). Fenilcetonúria no Sistema Único de Saúde: Avaliação de adesão ao
tratamento em um Centro de Atendimento do Rio Grande do Sul. Rev HCPA.

[B34] Palomo T, Fassier F, Ouellet J, Sato A, Montpetit K, Glorieux FH, Rauch F (2015). Intravenous bisphosphonate therapy of young children with
osteogenesis imperfecta: Skeletal findings during follow up throughout the
growing years. J Bone Miner Res.

[B35] Phillipi CA, Remmington T, Steiner RD (2008). Bisphosphonate therapy for osteogenesis
imperfecta. Cochrane Database Syst Rev.

[B36] Plotkin H, Rauch F, Bishop NJ, Montpetit K, Ruck-Gibis J, Travers R, Glorieux FH (2000). Pamidronate treatment of severe osteogenesis imperfecta in
children under 3 years of age. J Clin Endocrinol Metab.

[B37] Rauch F, Plotkin H, Zeitlin L, Glorieux FH (2003). Bone mass, size, and density in children and adolescents with
osteogenesis imperfecta: Effect of intravenous pamidronate
therapy. J Bone Miner Res.

[B38] Rauch F, Munns CF, Land C, Cheung M, Glorieux FH (2009). Risedronate in the treatment of mild pediatric osteogenesis
imperfecta: A randomized placebo- controlled study. J Bone Miner Res.

[B39] Rauch F, Travers R, Glorieux FH (2017). Pamidronate in children with osteogenesis imperfecta?:
Histomorphometric effects of long-term therapy. J Clin Endocrinol Metab.

[B40] Roughley PJ, Rauch F, Glorieux FH (2003). Osteogenesis imperfecta-clinical and molecular
diversity. Eur Cell Mater.

[B41] Sambrook P, Cooper C (2006). Osteoporosis. Lancet.

[B42] Shapiro JR, Germain-Lee EL (2012). Osteogenesis imperfecta: Effecting the transition from adolescent
to adult medical care. J Musculoskelet Neuronal Interact.

[B43] Sillence DO, Senn A, Danks DM (1979). Genetic heterogeneity in osteogenesis imperfecta. J Med Genet.

[B44] Thomas IH, DiMeglio LA (2016). Advances in the classification and treatment of osteogenesis
imperfecta. Curr Osteoporos Rep.

[B45] Trejo P, Rauch F (2016). Osteogenesis imperfecta in children and adolescents — new
developments in diagnosis and treatment. Osteoporos Int.

[B46] Trejo P, Fassier F, Glorieux FH, Rauch F (2017). Diaphyseal femur fractures in osteogenesis imperfecta:
Characteristics and relationship with bisphosphonate
treatment. J Bone Miner Res.

[B47] Van Dijk FS, Sillence DO (2014). Osteogenesis imperfecta: Clinical diagnosis, nomenclature and
severity assessment. Am J Med Genet A.

[B48] Ward LM, Rauch F, Whyte MP, D’Astous J, Gates PE, Grogan D, Lester EL, McCall RE, Pressly TA, Sanders JO (2011). Alendronate for the treatment of pediatric osteogenesis
imperfecta: A randomized placebo-controlled study. J Clin Endocrinol Metab.

[B49] Zambrano MB, Brizola ES, Refosco L, Giugliani R, Félix TM (2014). Anthropometry, nutritional status, and dietary intake in
pediatric patients with osteogenesis imperfecta. J Am Coll Nutr.

[B50] Zerbini CAF, Pippa MGB, Eis SR, Lazaretti-Castro M, Mota H, Tourinho TF (2007). Densitometria clínica: Posições oficiais 2006. Rev Bras Reumatol.

